# Genome modification of *CXCR4* by *Staphylococcus aureus* Cas9 renders cells resistance to HIV-1 infection

**DOI:** 10.1186/s12977-017-0375-0

**Published:** 2017-11-15

**Authors:** Qiankun Wang, Shuliang Chen, Qiaoqiao Xiao, Zhepeng Liu, Shuai Liu, Panpan Hou, Li Zhou, Wei Hou, Wenzhe Ho, Chunmei Li, Li Wu, Deyin Guo

**Affiliations:** 1 School of Basic Medical Sciences, Wuhan University, Wuhan, 430072 People’s Republic of China; 2 Center for Retrovirus Research, Department of Veterinary Biosciences, The Ohio State University, 1900 Coffey Road, Columbus, OH USA; 3 College of Life Sciences, Wuhan University, Wuhan, People’s Republic of China; 4 Animal Biosafety Level III Laboratory at the Center for Animal Experiment, Wuhan University, Wuhan, People’s Republic of China; 5 School of Medicine (Shenzhen), Sun Yat-sen University, Zhongshan Erlu 74, Yuexiu District, Guangzhou, 510080 People’s Republic of China

**Keywords:** CXCR4, HIV-1, Primary CD4^+^ T cells, Adeno-associated virus, CRISPR/SaCas9

## Abstract

**Background:**

The CRISPR/Cas9 system has been widely used for genome editing in mammalian cells. CXCR4 is a co-receptor for human immunodeficiency virus type 1 (HIV-1) entry, and loss of *CXCR4* function can protect cells from CXCR4 (X4)-tropic HIV-1 infection, making *CXCR4* an important target for HIV-1 gene therapy. However, the large size of the CRISPR/SpCas9 system presents an obstacle to its efficient delivery into primary CD4^+^ T cells. Recently, a small *Staphylococcus aureus* Cas9 (SaCas9) has been developed as a genome editing tool can address this question. Therefore, it provides a promising strategy for HIV-1 gene therapy if it is used to target CXCR4.

**Results:**

Here, we employed a short version of Cas9 from *Staphylococcus aureus* (SaCas9) for targeting *CXCR4*. We demonstrated that transduction of lenti-virus expressing SaCas9 and selected single-guided RNAs of *CXCR4* in human CD4^+^ T cell lines efficiently induced the editing of the *CXCR4* gene, making these cell lines resistant to X4-tropic HIV-1 infection. Moreover, we efficiently transduced primary human CD4^+^ T cells using adeno-associated virus-delivered CRISPR/SaCas9 and disrupted CXCR4 expression. We also showed that *CXCR4*-edited primary CD4^+^ T cells proliferated normally and were resistant to HIV-1 infection.

**Conclusions:**

Our study provides a basis for possible application of *CXCR4*-targeted genome editing by CRISPR/SaCas9 in HIV-1 gene therapy.

**Electronic supplementary material:**

The online version of this article (10.1186/s12977-017-0375-0) contains supplementary material, which is available to authorized users.

## Background

Human immunodeficiency virus type 1 (HIV-1) is the causative agent of acquired immunodeficiency syndrome (AIDS). According to WHO, over 2.1 million people were newly infected with HIV-1 in 2015 with an estimated 1.1 million people dying of AIDS-related illnesses in the same year, and currently over 36.7 million people worldwide are HIV-1-positive (http://www.who.int/en/). The use of antiretroviral therapy (ART) limits HIV-1 replication to an undetectable level, thus improving the health and life expectancy of HIV-1 infected individuals. However, ART does not represent a complete cure for HIV-1 infection as it is not effective against a persistent latent viral reservoir and has several side effects [1, 2]. Thus, new curative approaches with limited drawbacks and higher efficiency for the treatment of HIV/AIDS are urgently needed.

HIV-1 entry into target cells is initiated by binding of the viral envelope protein gp120 to the primary receptor CD4 and a major co-receptor, either CXCR4 or CCR5 [3, 4]. CCR5 is the co-receptor required for the entry of CCR5 (R5)-tropic strains of HIV-1, while CXCR4 (X4)-tropic viral strains are dependent on the CXCR4 co-receptor. Individuals with a naturally homozygous *CCR5Δ32* deletion are highly resistant to HIV-1 infection [5, 6]. Furthermore, previous studies reported a functional cure of HIV-1 infection when an AIDS patient with leukemia received a bone-marrow transplant from a tissue-matched donor with homozygous *CCR5Δ32* mutation [7, 8]. Thus, the co-receptor CCR5 has been the major target for genome editing against HIV-1 infection. However, X4-tropic HIV-1 strains emerge in nearly a half of the patients initially infected with R5-tropic HIV-1 and their emergence is associated with a faster disease progression [9, 10]. Therefore, CXCR4 should be considered another important target for anti-HIV-1 gene therapy.

Over the last decade, novel genome-editing methods that utilize nucleases have been developed, including zinc finger nucleases (ZFNs) [11], transcription activator like-effector nucleases (TALENs) [12] and clustered regularly interspaced short palindromic repeats (CRISPR)/ CRISPR-associated nuclease (Cas9) [13, 14]. Disruption of *CXCR4* by ZFN-mediated genome editing conferred resistance to X4-tropic HIV-1 in several studies. Wilen et al. showed that disruption of *CXCR4* with ZFNs conferred resistance of human CD4^+^ T cells to X4-tropic HIV-1 strains [15]. Yuan et al. showed that disruption of *CXCR4* with ZFNs in human CD4^+^ T cells provided protection from HIV-1 infection in tissue cultures and in NSG mice [16]. Using the same approach, Didigu et al. showed that simultaneous genetic modification of *CCR5* and *CXCR4* in primary human CD4^+^ T cells rendered cells resistant to infection with R5- and X4-tropic HIV-1 strains in vitro and in vivo [17].

CRISPR/Cas9 offers several advantages over conventional ZFN and TALEN, such as simple to design, easy to use and multiplexing [18]. Hultquist et al. edited the *CXCR4* or *CCR5* gene in primary CD4^+^ T cells by electroporation of CRISPR/Cas9 ribonucleoproteins [19]. We previously showed that the first generation of CRISPR/SpCas9 system was able to disrupt *CXCR4* in primary human CD4^+^ T cells and generate HIV-1 resistance [20]. However, the large size of the CRISPR/SpCas9 system restricts its efficient delivery into primary CD4^+^ T lymphocytes. Li et al. used a chimeric adenovirus as a vector for the delivery of CRISPR/SpCas9, which resulted in the efficient silencing of *CCR5* and, thus, HIV-1 resistance in primary CD4^+^ T cells [21]. In contrast, Wang et al. showed that lentiviral vectors expressing SpCas9 and sgRNA efficiently disrupt the *CD4*, *CCR5* and *CXCR4* genes in transduced human CD4^+^ T cell line, but not in primary human CD4^+^ T cells [22].

One of the major challenges for CRISPR/Cas9 gene editing technologies is the delivery efficiency of the large gene cassettes. Viral vectors that including lentivirus, adenovirus, adeno-associated virus (AAV) are potential delivery vehicles for CRISPR/Cas9 components [23, 24]. AAV capsids can package less than 4.7 kb of single-stranded DNA, leaving little room for inserting other genetic elements when adopting the widely used Cas9 from *Streptococcus pyogenes* (SpCas9, 4.2 kb). The Cas9 from *Staphylococcus aureus* (SaCas9) is 1 kb shorter than SpCas9 and thus can be packaged into the AAV genome together with a sgRNA gene expression cassette. Moreover, SaCas9 has a longer protospacer-adjacent motif (PAM) of 5′-NNGRRT-3′ sequence compared to SpCas9 PAM of 5′-NGG-3′. These features allow easier delivery to cells by AAV expression vectors, and higher sequence specificity, which would be more desirable for therapeutic applications [25]. AAV-mediated SaCas9/sgRNA could be used to excise the integrated HIV-1 genome in vivo [26, 27]. Using AAV as a gene therapy vector has many advantages over other commonly used recombinant viral vectors, such as low toxicity, sustained gene expression, safe and efficient delivery [28]. Recent studies reported that use of AAV6 in combination with electroporation of nuclease mRNA results in efficient homologous recombination repair (HDR) -mediated genome editing in transduced primary T cells and CD34^+^ hematopoietic cells [29–31]. We thus sought to deliver the CRISPR/SaCas9 into primary CD4^+^ T cells using an AAV6 vector.

In the present study, we selected two SaCas9/sgRNAs with high specificity to target *CXCR4* after screening of 12 sgRNAs in HEK293T cell lines by using lentiviral vectors. By utilizing an AAV vector, we demonstrated the efficacy of *CXCR4* editing and established HIV-1 resistance in primary CD4^+^ T cells.

## Results

### Disruption of *CXCR4* in GHOST-X4 and TZM-bl cells by lentiviral vector-mediated transduction renders cells resistant to HIV-1 infection

**Fig. 1:**
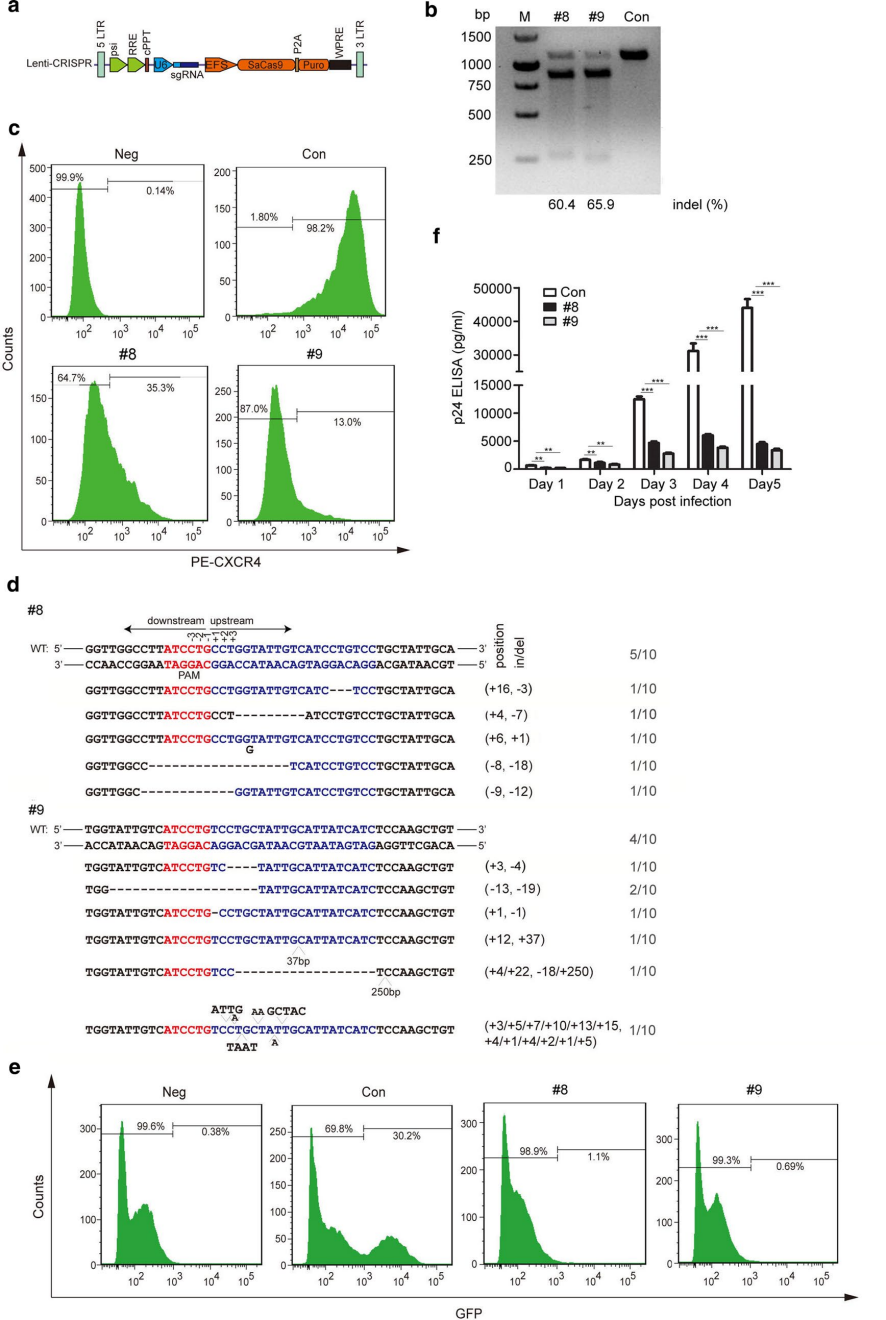
*CXCR4* gene silencing by lentivirus mediated CRISPR/SaCas9 delivery protects GHOST-X4 cells from HIV-1 infection. **a** A schematic diagram of lentiviral transfer vectors containing CRISPR/SaCas9 components. LentiCRISPR v2 plasmid was modified by replacing the SpCas9 with SaCas9. Then, based on the SaCas9 PAM sequence 5′-NNGRRT-3′, we designed, synthesized and cloned the CXCR4 sgRNAs into the vector using the *Bsmb1*. **b** *CXCR4* gene disruption analysis in GHOST-X4 cells by the T7E1 cleavage assay. GHOST-X4 cells were transduced with lentiviruses (MOI of 40) in the presence of polybrene for 12 h, and the genomic DNA was extracted and used as template to amplify a *CXCR4* fragment (1100 bp). Con: lentiviral vectors expressing SaCas9 only, #8 and #9: lentiviral vectors expressing SaCas9/sgRNA #8 and #9. **c** Flow cytometry analysis of CXCR4 expression in lentivirus transduced GHOST-X4 cells. Neg, unstained cells. Con, #8 and #9 as samples described in (**b**) stained with anti-CXCR4-PE. **d** DNA sequences of *CXCR4* of the transduced GHOST-X4 cells. PCR products were cloned into pGEM-T Easy vector and sequenced. The PAM sequences are lined and highlighted in red; the target sequences were shown in blue; deletions are indicated with (−) and insertions with (+). N/N indicates ratio of WT or mutations to total sequenced clones. **e** Flow cytometry analysis of transduced GHOST-X4 cells on GFP expression 3 days post HIV-1_NL4-3_ infection. Neg, no HIV-1 infection. **f** HIV-1_NL4-3_ infection was determined by p24 level. The cultured supernatants were collected at the indicated days post infection, and HIV-1 p24 level was determined using a p24 ELISA kit. For (**c** and **e**), one representative out of three independent experiments is shown. For (**f**), the graph represents 3 independent infection experiments and error bars represent SEM. Statistical analysis determined using unpaired t-test (***P < 0.001; **P < 0.01; *P < 0.05)

To silence *CXCR4* expression, we designed 12 sgRNAs that target different sites within the *CXCR4* exon 2 and CCR5 sgRNA as the negative control (Additional file 1: Table S1). In order to select the efficient target sites, we modified the lentiCRISPR v2 plasmid by replacing the SpCas9 with SaCas9, and cloned the sgRNAs into the vector (Fig. 1a). We first tested the ability of our modified lentiCRISPR/SaCas9 gene editing system to disrupt *CXCR4* in HEK293T cells. Three days after the transfection, we tested the on-target efficacy of the SaCas9/sgRNAs using a T7E1 assay, which cleaves DNA at distorted duplexes caused by mismatches. The result showed that the 1100-bp PCR amplicon was cleaved by sgRNA #4, #8, #9 #10 and #12 guided SaCas9 while the negative sgRNA (sg-CCR5) guided no detectable editing (Additional file 2: Figure S1). We also observed that sgRNA #8 and #9 exhibited much higher efficiency than other sgRNAs (Additional file 2: Figure S1).

We subsequently tested the editing efficacy of the sgRNA #8 and #9 to disrupt *CXCR4* in GHOST-X4 reporter cells, which express high levels of CD4 and CXCR4, and harbor a stable promoter element of long terminal repeat (LTR) -GFP reporter. Upon HIV-1 infection, viral Tat protein will bind to the LTR promoter and activate GFP expression [32]. The lentiCRISPR/SaCas9 vector was transduced into GHOST-X4 cells, and 3 days after the transduction, we tested the on-target efficacy of the SaCas9/sgRNAs using a T7E1 assay. The result showed that the 1100-bp PCR amplicon was efficiently cleaved into two fragments by SaCas9/sgRNAs #8 and #9, leading to disruption of the *CXCR4* gene (Fig. 1b). We also measured the expression of CXCR4 protein on the cell surface by flow cytometry. As shown in Fig. 1c, GHOST-X4 cells transduced with lentiviral vectors expressing SaCas9/sgRNA #8 and #9 became 64.7 and 87.0% negative for CXCR4 surface expression, respectively. In contrast, no reduction of CXCR4 expression was observed in cells transduced with empty vector (as a negative control). The sequencing results revealed that SaCas9/sgRNA #8 and #9 efficiently induced indel mutations in *CXCR4* gene (Fig. 1d). We next tested the effect of the *CXCR4* gene disruption on X4-tropic HIV-1_NL4-3_ infection. We found that GHOST-X4 cells transduced with lentiviral SaCas9/sgRNA #8 or #9 became negative for GFP expression compared to control cells (Fig. 1e), indicating that lentiCRISPR/SaCas9-mediated genome modification of CXCR4 protects GHOST-X4 cells from HIV-1 infection. Furthermore, HIV-1 infection was determined by measurement of viral p24 in the culture supernatants. The levels of p24 released from the transduced cells were significantly decreased compared to the control cells (Fig. 1f).

**Fig. 2:**
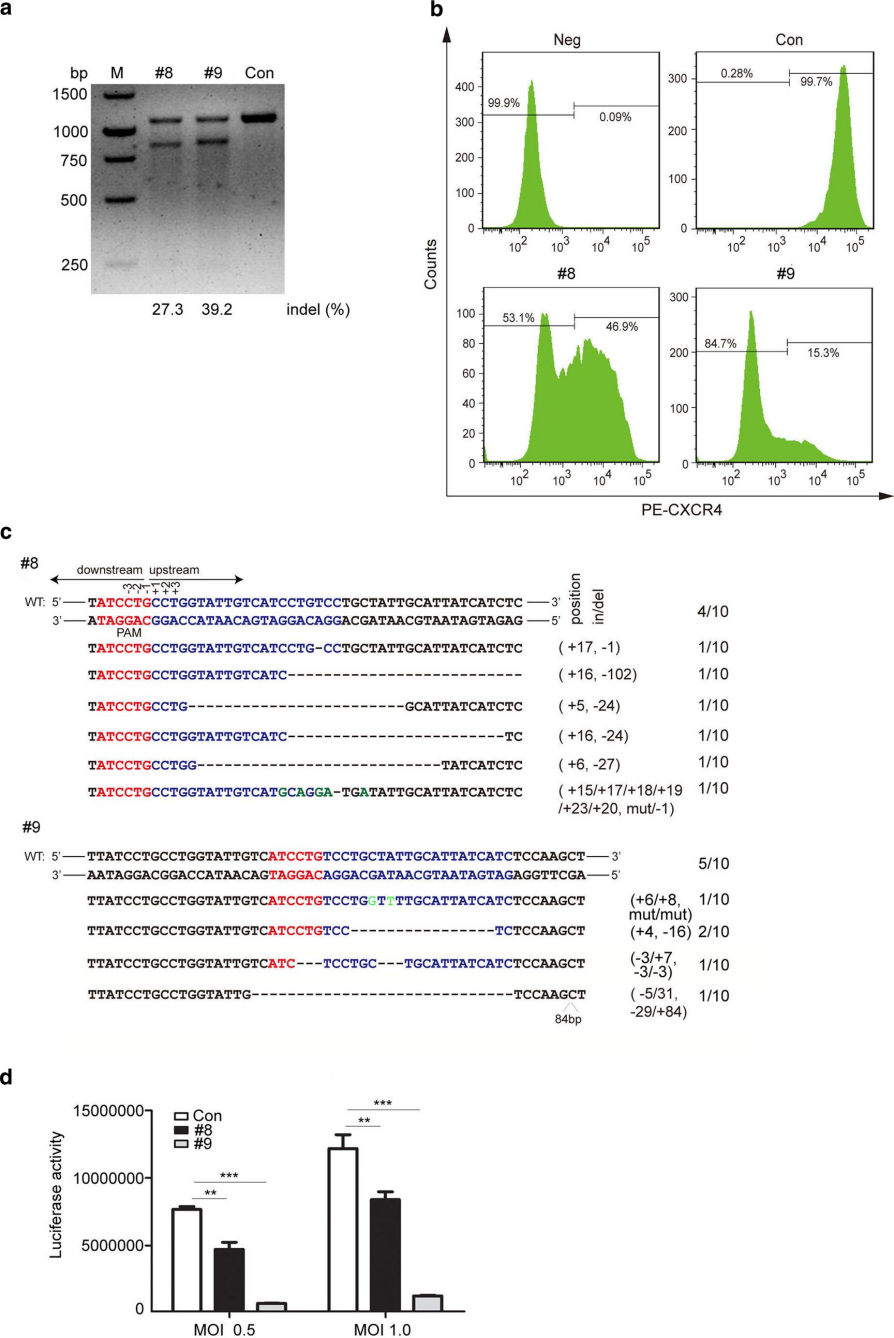
Disruption of *CXCR4* in TZM-bl cells via lentiCRISPR/SaCas9 renders cells resistant to HIV-1 challenge. **a** *CXCR4* gene disruption analysis in TZM-bl cells by the T7E1 cleavage assay. Assays were performed as in Fig. 1b. **b** Flow cytometry analysis of CXCR4 expression in lentivirus transduced TZM-bl cells. Assays were performed as in Fig. 1c. **c** DNA sequences of *CXCR4* of the transduced TZM-bl cells. Assays were performed as in Fig. 1d. **d** Luciferase reporter assay to quantify HIV-1 infection level. Modified TZM-bl cells were infected with HIV-1_NL4-3_ (MOI of 0.5 or 1) for 6 h, then washed three times with PBS and cultured in complete DMEM medium for 3 days. Cells were collected and lysed in 100 µl of lysis buffer (Promega) for luciferase assay. For (**b**), one representative out of three independent experiments is shown. For (**d**), the graph represents 3 independent infection experiments and error bars represent SEM. Statistical analysis determined using unpaired t-test (***P < 0.001; **P < 0.01; *P < 0.05)

Similarly, CXCR4 expression on another HIV-1 reporter cell line TZM-bl was also disrupted by SaCas9/sgRNA #8 and #9 (Fig. 2a–c). TZM-bl cells are originally derived from HeLa cells expressing human CD4, CXCR4 and CCR5 as well as HIV-1 LTR-driven reporter genes including *firefly luciferase* [33]. SaCas9/sgRNA (#8 and #9) modified TZM-bl cells were infected with replication-competent HIV-1_NL4-3_ followed by luciferase reporter assay to quantify virus infection. Results revealed that HIV-1_NL4-3_ infection in modified cells was largely decreased relative to the control cells, and SaCas9/sgRNAs #9 showed higher disruption efficiency (Fig. 2d). Together, these data suggested that disruption of *CXCR4* in HIV-1 reporter cell lines by lentivirus transduction renders cells resistant to X4-tropic HIV-1 infection.

### *CXCR4* disruption by SaCas9/sgRNA in lentiviral vector-transduced Jurkat T cells inhibits HIV-1 infection

**Fig. 3:**
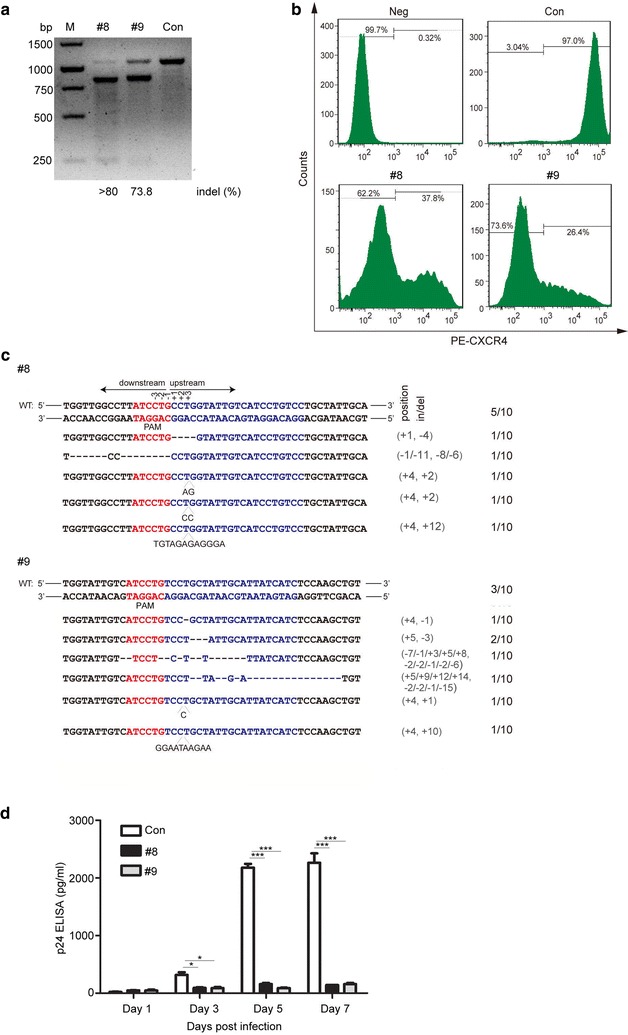
Genome editing of *CXCR4* in Jurkat T cells confers cell inhibition to HIV-1 infection by lentiCRISPR/SaCas9. **a** *CXCR4* gene disruption analysis in Jurkat T cells by T7E1 cleavage assay. Assays were performed as in Fig. 1b. **b** Flow cytometry analysis of CXCR4 expression in lentivirus transduced Jurkat T cells. Assays were performed as in Fig. 1c. **c** DNA sequences of *CXCR4* of the transduced Jurkat T cells. Assays were performed as in Fig. 1d. **d** HIV-1 p24 was detected in the Jurkat T cells treated with SaCas9/sgRNA. For (**b**), one representative out of three independent experiments is shown. For (**d**), the graph represents 3 independent infection experiments and error bars represent SEM. Statistical analysis determined using unpaired *t* test (***P < 0.001; **P < 0.01; *P < 0.05)

To test the effect of CRISPR/SaCas9-mediated disruption of *CXCR4* in CD4^+^ T cell lines, we transduced Jurkat T cells with lentiviral vectors encoding SaCas9/sgRNA #8 and #9. T7E1 assay and sequencing showed that the indels of *CXCR4* gene were induced by SaCas9/sgRNA (Fig. 3a, c). We also performed flow cytometry to analyze CXCR4 protein expression and confirmed that cell surface CXCR4 expression was decreased by 62.2 and 73.6% using SaCas9/sgRNA #8 and #9, respectively (Fig. 3b). We further assessed the ability of SaCas9/sgRNAs to suppress HIV-1_NL4-3_ infection of Jurkat T cells by measuring viral p24 levels in culture supernatants at 1–7 days post-infection. Compared to the control cells, a significant decrease in p24 levels was observed in the Jurkat T cells treated with two SaCas9/sgRNAs (Fig. 3d). These data demonstrated that Jurkat T cells with disrupted *CXCR4* gene by SaCas9/sgRNA are highly resistant to HIV-1 infection.

### *CXCR4* editing protects primary CD4^+^ T cells from HIV-1 infection by AAV-CRISPR/SaCas9

**Fig. 4:**
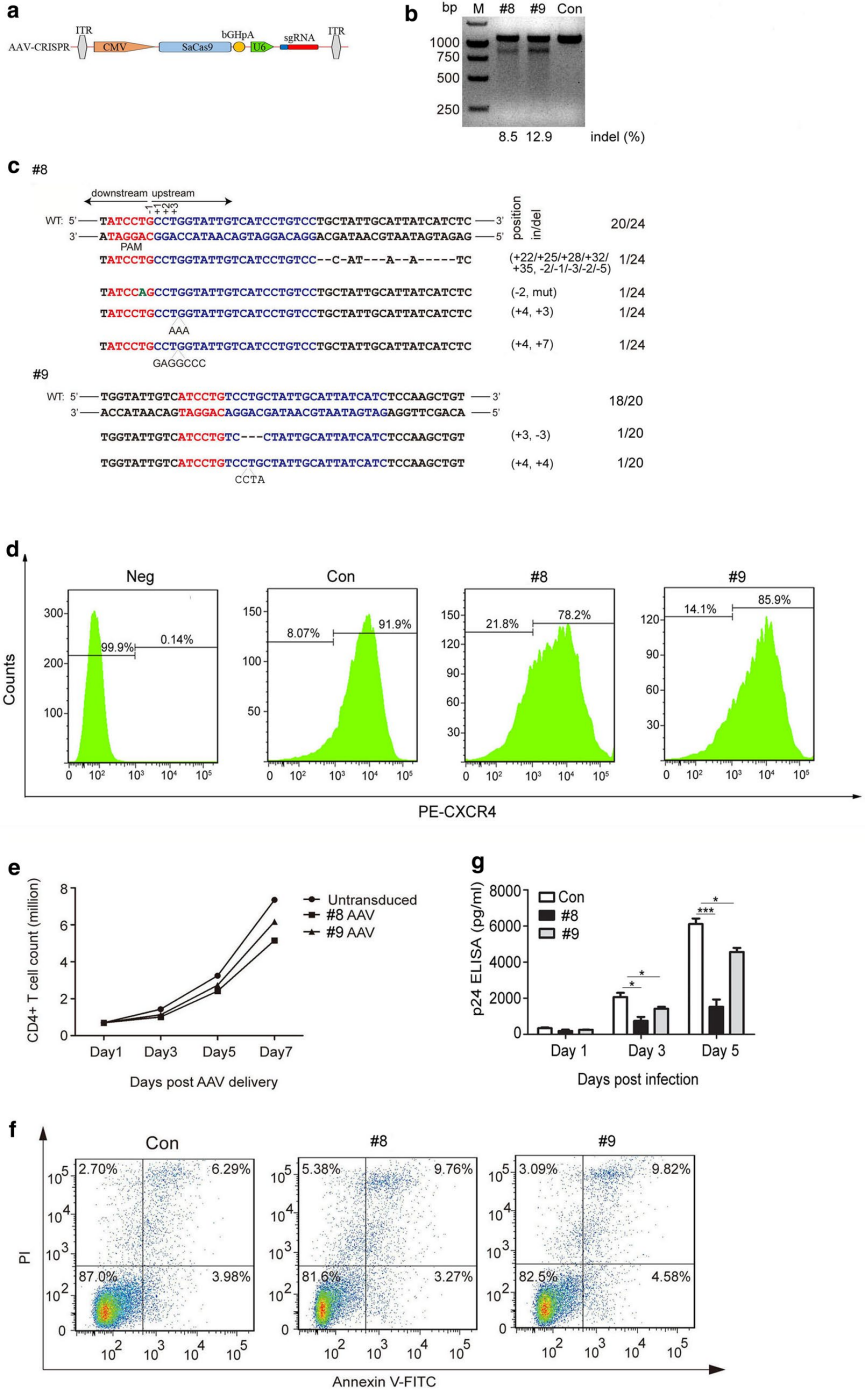
AAV-mediated SaCas9/sgRNAs delivery suppresses HIV-1 infection in human primary CD4^+^ T cells. **a** A schematic diagram of AAV transfer vectors containing SaCas9 endonuclease and sgRNA. **b** T7E1 cleavage assay after 5 days post-transduction. **c** DNA sequences of *CXCR4* of the AAV transduced CD4^+^ T cells. **d** Flow cytometry analysis of CXCR4 expression in AAV transduced CD4^+^ T cells. Assays were performed as in Fig. 1c. **e** CD4^+^ T cells counts at different times after transduction. CXCR4 disrupted cells continued to grow normally. **f** Flow cytometry analysis of apoptosis following AAV delivery SaCas9/sgRNA. **g** HIV-1 p24 was detected in the supernatants of the CD4^+^ T cells treated with AAV delivered SaCas9/sgRNA following HIV-1_NL4-3_ infection. For (**d**, **e** and **f**), one representative out of three independent experiments is shown. For (**g**), the graph represents 3 independent infection experiments and error bars represent SEM. Statistical analysis determined using unpaired t-test (***P < 0.001; **P < 0.01; *P < 0.05)

After successfully disrupting *CXCR4* in cell lines by lentiviral vectors expressing SaCas9/sgRNAs, we attempted to deliver the CRISPR/SaCas9 components into primary CD4^+^ T cells. Despite our best efforts, we were not able to disrupt *CXCR4* in primary human CD4^+^ T from multiple donors by lentiviral vectors expressing SaCas9/sgRNAs. We thus sought to deliver the CRISPR/SaCas9 into primary CD4^+^ T cells using an AAV vector. We constructed AAV vectors harboring the expression cassettes of SaCas9 and sgRNA #8 or #9, respectively (Fig. 4a). To evaluate the efficiency of AAV constructs for genome editing in primary CD4^+^ T cells, we performed the T7E1 assay and sequencing at 5 days post-transduction. Cells transduced with AAV-SaCas9/sgRNA #8 and #9 showed efficient cleavage of the C*XCR4* gene compared with the control cells transduced with AAV expressing only SaCas9 (Fig. 4b, c). We also used electroporation to deliver the SaCas9/sgRNA #8 and #9 into CD4^+^ T cells and observed disruption and indels in *CXCR4* (Additional file 3: Figure S2). We next performed flow cytometry to analyze CXCR4 protein expression. CD4^+^ T cells transduced with AAV vectors expressing SaCas9/sgRNA #8 and #9 became 21.8 and 14.1% negative for CXCR4 surface expression, respectively. As a control, 8.07% negative for CXCR4 expression was observed in cells transduced with empty vector (Fig. 4d). To examine whether *CXCR4* silencing could affect cell viability, we performed a cell-viability assay at 1–7 days post-transduction and analyzed apoptosis upon AAV-mediated delivery of SaCas9/sgRNAs. The early apoptosis rate of SaCas9/sgRNA #8 and #9-transduced cells was 3.27 and 4.58%, respectively, compared with 3.98% of control cells (Fig. 4f). These results revealed that *CXCR4* editing did not significantly affect cell growth compared with control cells (Fig. 4e and f). Next, we assessed whether the editing of the *CXCR4* could render cells resistant to replication-competent HIV-1_NL4-3_ infection. The AAV-transduced CD4^+^ T cells were infected with HIV-1_NL4-3_ and the culture supernatants were collected at 1–5 days post-infection for p24 detection. The level of p24 from CD4^+^ T cells transduced with SaCas9/sgRNA #8 or #9 was markedly decreased compared with that of cells transduced with empty vector at 3–5 days post-infection (Fig. 4g). These results suggested that the AAV-SaCas9/sgRNA system can be used to mediate efficient editing of *CXCR4* in primary CD4^+^ T cells and confer resistance to HIV-1 infection, while not affecting cell growth and viability.

### Specific targeting of *CXCR4* by CRISPR/SaCas9 does not induce detectable off-target effects

The off-target sites of CRISPR/SaCas9 were predicted and aligned with human genome using the online tool Cas-OFFinder (http://www.rgenome.net/cas-offinder/) [34]. We extracted 9 candidate off-target sites (Additional file 4: Table S2), and the sequences of these predicted off-target sites were PCR amplified and subjected to T7E1 assay to identify the off-target activity. We did not detect any mutations using T7E1 assay (Additional file 5: Figure S3), indicating that CRISPR/SaCas9 showed high specificity of gene editing in our experiments.

## Discussion

Currently available ART drugs do not provide an effective cure for HIV-1 infection. Gene therapy may represent a valuable alternative approach for AIDS treatment. One approach with ZFN-CCR5-modified autologous T cells of HIV-infected subjects is evaluated in an ongoing Phase 2 trial (SB-728) [35]. Although HIV-1 strains use CCR5 as a major coreceptor when establishing initial infections, CXCR4 is also a coreceptor for CXCR4-tropic HIV-1 infection in vivo [9, 10]. Therefore, disruption of CXCR4 expression can complement CCR5-specific therapy against HIV-1 infection.

In our previous report, the SpCas9 system has been successfully used to disrupt CXCR4 in GHOST-X4 and Jurkat T cells by lenti-SpCas9/sgRNA with efficiency of 76.5 and 45.1% respectively [20]. In the parental assay, the efficiency of lenti- SaCas9/sgRNA mediated *CXCR4* editing of GHOST-X4 and Jurkat T cells are 87.0 and 73.6%. These data suggested that SaCas9-mediated CXCR4 genome editing has more potential than SpCas9. Our previous results showed that editing of *CXCR4* in primary human CD4^+^ T cells by delivery of SpCas9/sgRNA utilizing nucleofection generates HIV-1 resistance [20]. Although nucleofection is a direct delivery approach, its low efficacy and toxicity to primary cells restrict its application. In this study, we employed a short version of Cas9 from *Staphylococcus aureus* and AAV delivery system for targeting *CXCR4*. AAV can transduce both dividing and non-dividing cells without integrating into the host genome and shows low toxicity and sustained gene expression, while being safe in human gene therapy [23, 25]. We confirmed that primary human CD4^+^ T cells could be efficiently transduced and edited by AAV-delivered CRISPR/SaCas9 targets CXCR4. Thus, our report provides an alternative approach for disrupting *CXCR4* for HIV-1 therapy.

As CXCR4 is the receptor for CXCL12 (SDF-1) and has important consequence for T cell function and trafficking [36], we should pay attention to the safety and side effects before clinical application by targeting CXCR4 in HIV-1 gene therapy. However, studies showed that T lymphocytes appear to develop normally in *CXCR4* knockout mice, suggesting that disruption of *CXCR4* in CD4^+^ T cells may be tolerable [37, 38]. Although debatable, disruption of *CXCR4* by ZFN in several studies demonstrated that CXCR4 is not essential for CD4^+^ T cell viability and function [15–17]. In our previous two reports, *CXCR4* disruption by CRISPR-SpCas9 did not cause cells viability in CD4^+^ T cell [20, 39]. In this study, we also showed that CXCR4-edited primary CD4^+^ T cells proliferated normally. In addition, we found that sgRNA #9 works better than sgRNA#8 in primary cells, while opposite effect was observed in the cell lines. This probably resulted from various efficacies of expression and activity of promoter between EFS-NS (lenti-CRISPR/Cas9) and CMV (AAV-CRISPR/Cas9) in different cells, or other underlying mechanisms [40].

## Conclusions

In summary, our results demonstrated that *CXCR4* disruption in CD4^+^ T cell lines using lentiviral vectors expressing SaCas9/sgRNAs results in resistance to HIV-1 infection. Moreover, we showed that transducing AAV6 expressing SaCas9/sgRNAs in primary CD4^+^ T cells confers protection to HIV-1 infection. Together, this work represents a proof-of-principle study and provides a potential alternative approach for HIV-1/AIDS genetic therapy by targeting CXCR4 with AAV-deliver SaCas9/sgRNAs.

## Methods

### sgRNA design and plasmid construction

Based on the SaCas9 PAM sequence 5′-NNGRRT-3′, 12 gRNAs targeting CXCR4 exon 2 were designed and synthesized with 5′-CACC and 5′-AAAC overhangs. For lenti-SaCas9-CXCR4-gRNA plasmid, we modified the lentiCRISPR v2 plasmid (Addgene #52961) by replacing the SpCas9 with SaCas9, and cloned the sgRNAs into the vector using the *Bsmb1* (Fermentas). For AAV-SaCas9-CXCR4-gRNA plasmid, sgRNAs were inserted into the PX601 plasmid (Addgene #61591) digested with *Bsa1* (Fermentas). Oligonucleotides for sgRNAs targeting *CXCR4* are shown in Additional file 1: Table S1.

### Cell culture and transfection

HEK293T cells, GHOST-X4 cells, TZM-bl and Jurkat T cells were cultured as described previously [41]. HEK293T cells were transfected in 24-well plates using 1 µg of plasmid DNA mixed with Polyethylenimine (PEI) (Polysciences) according to the manufacturer’s instruction.

### Primary CD4^+^ T cell isolation and electroporation

Whole blood samples from healthy donors were purchased from the Wuhan Blood Center (Wuhan, China). Isolation of CD4^+^ T cells was achieved using Miltenyi products (Miltenyi Biotech). Primary human CD4^+^ T cells were maintained in RPMI (Hyclone) supplemented with 10% FBS (Gibco), 1% penicillin/streptomycin (Hyclone), 20 ng/ml IL-2 (PeproTech) and activated with anti-CD3/CD28-beadscoated on culture plate (Biolegend). Cells were seeded at 1–2 × 10^6^/ml and cultured in T25 flasks.

CD4^+^ T cells were electroporated using the Lonza Nucleofector 4D (program E0-115) and the P3 Primary Cell 4D-Nucleofector Kit (V4XP-3024). In brief, 5 × 10^6^ CD4^+^ T cells were collected and washed twice in PBS. The cells were resuspended in 100 µl Nucleofector Solution with 5 µg of AAV-CRISPR/SaCas9 plasmids respectively. The mixture was transferred into the Nucleocuvette vessel and electro-transfecte. Following electroporation, the cells were cultured in RPMI 1640 medium supplemented with 10% FBS.

### Virus production and infection

Lentiviruses were produced by co-transfection of HEK293T cells with lenti-SaCas9-CXCR4-gRNA, psPAX2 and pMD2.G, followed by concentration of the virus stocks by ultracentrifugation. Lentiviruses and HIV-1_NL4-3_ stocks were generated as described before [20]. GHOST-X4 cells, TZM-bl cells and Jurkat T cells were transduced with lentiviruses (MOI (multiplicity of infection) of 40) in the presence of 8 µg/ml polybrene (Sigma) for 12 h, and then medium was changed. For Jurkat T cells, the 12-well plate were spun at 1200 g for 1.5 h at 25 °C then transferred to 37 °C. 36 h post-transduction, 1 µg/ml puromycin (Sigma) was added to kill untransduced TZM-bl and Jurkat T cells within 2 days. After removal of puromycin, transduced cells were further cultured until downstream analysis. AAV were purchased from Vigene Biosciences (Jinan, China). CD4^+^ T cells were cultured with IL-2 and activated with anti-CD3/CD28-beads coated on culture plate for 24–36 h and then transduced with AAV vectors at 5 × 10^5^ vector genome copy per cell in free-FBS media for 24 h. The cells were washed 3 times with PBS then resuspended in fresh media.

### T7 endonuclease 1 (T7E1) cleavage assay and DNA sequencing

Genomic DNA was extracted with the Blood and Cell Culture DNA Midi kit (TianGen, China) according to the manufacturer’s instructions. The purified genomic DNA was used as a template to amplify a fragment of the *CXCR4* gene using the specific primers. The fragment size was 1100 bp (Additional file 6: Table S3). The PCR products were digested with T7 endonuclease 1 (NEB) and resolved by 1.5% agarose gel electrophoresis. The densities of cut and uncut bands were calculated using Image J software. We used ${\text{Indel}}\,(\%)= \left( {1 - \sqrt {1 - {\text{cut}}/{\text{uncut}} + {\text{cut}}} } \right) \times 100\%$ formulas to get the efficiency of cleavage activity. The T7EI cleaves DNA at distorted duplexes with an upper limit of disruption sensitivity 80% [42]. To further analyze *CXCR4* gene disruption, the above PCR products were cloned into pGEM-T Easy vector (Promega) and sequenced. The indels of the *CXCR4* gene were identified by comparison with the wild-type *CXCR4* sequence.

### Flow cytometry analysis

To analyze cell surface expression of CXCR4, lentivirus transduced GHOST-X4 cells, TZM-bl cells and Jurkat T cells were incubated with a PE-conjugated mouse anti-human CXCR4 antibody (Biolegend) for 20 min on ice. Cells then were washed twice with PBS and analyzed by flow cytometry (FACS AriaIII, BD). To determine HIV-1 infection efficiency, modified GHOST-X4 cells were infected with HIV-1_NL4-3_ at an MOI of 1 for 6 h, then washed three times with PBS and cultured in complete DMEM medium for 3 days. GHOST-X4 cells were collected and washed twice with PBS and assessed for GFP expression through flow cytometry (FACS AriaIII, BD). Data were analyzed with FlowJo (TreeStar) software. To test cell viability, primary CD4^+^ T cells were collected after 5 days post AAV transduction, and 5 µl Annexin V-FITC and 5 µl propidium iodide (PI) (BD) were added and incubated for 15 min at room temperature. The apoptosis of AAV transduced cells were determined by flow cytometry (FACS AriaIII, BD).

### Luciferase activity assay and p24 detection by ELISA

Modified TZM-bl cells were infected with HIV-1_NL4-3_ at an MOI of 0.5 or 1 for 6 h, then washed three times with PBS and cultured in complete DMEM medium for 3 days. Cells were then washed once with PBS and lysed in 100 µl of lysis buffer (Promega). Luciferase activity in 30 µl of cell suspensions was measured by a BrightGlo Luciferase assay according to the manufacturer’s instruction (Promega). The cultured supernatants were collected at indicated days post-infection, HIV-1 p24 was determined using a p24 ELISA kit (ZeptoMetrix).

### Off-target site analysis

To analyze potential off-target mutations, we used an online platform Cas-OFFinder (http://www.rgenome.net/cas-offinder/) and allowed up to a 4 bp mismatch. The primers for amplifying the off-target sites resulted in 600-bp amplicons centered near the off-target sites. The corresponding primers are listed in Additional file 6: Table S3. The modified Jurkat T cells genomic DNA was used as a template to amplify the off-target sites. The T7E1 assay was used to detect off-target cleavage.

### Statistical analysis

Unpaired *t* test was performed using GraphPad Prism version 5. *P < 0.05, **P < 0.01, and ***P < 0.001 denote significant differences. All experiments were performed for at least three times.

## Additional files

**Additional file 1: Table S1.** Oligonucleotides for sgRNAs targeting *CXCR4* locus.

**Additional file 2: Figure S1.** *CXCR4* gene disruption screening analysis in HEK293T cells by the T7E1 cleavage assay. HEK293T cells were transfected in 24-well plates using 1 µg of plasmid DNA mixed with Polyethylenimine (PEI). Three days after the transfection, the genomic DNA was extracted and used as template to amplify a *CXCR4* fragment (1100 bp). Neg: CCR5 sgRNA; Con: lentiviral vectors expressing SaCas9 only; #1–#12: lentiviral vectors expressing SaCas9/sgRNA #1–#12.

**Additional file 3: Figure S2.** (a) T7E1 cleavage assay after electroporation in CD4^+^ T cells. (b) DNA sequences of *CXCR4* of electroporated CD4^+^ T cells.

**Additional file 4: Table S2.** List of potential off-target sites for both 5′ and 3′ sgRNAs.

**Additional file 5: Figure S3.** (a) Off-target analysis of CXCR4 (#8) by T7E1 cleavage assay. (b) Off-target analysis of CXCR4 (#9) by T7E1 cleavage assay.

**Additional file 6: Table S3.** Primers information for the study.
