# Supplementary material for: Genome modification of CXCR4 by Staphylococcus aureus Cas9 renders cells resistance to HIV-1 infection
Source: Retrovirology. 2017 Nov 15;14:51. doi: 10.1186/s12977-017-0375-0 (PMC5688617; doi:10.1186/s12977-017-0375-0)
Supplement: Supplementary file 1 — Additional file 1: Table S1. Oligonucleotides for sgRNAs targeting CXCR4 locus. [file 12977_2017_375_MOESM1_ESM.docx]

Table S1. Oligonucleotides for sgRNAs targeting *CXCR4* locus

| sgRNA | Targeting sequences-PAM (NNGRRT) | Oligonucleotide used to make sgRNA construct |
| --- | --- | --- |
| #1 | AATGGATTGGTCATCCTGGTCATGGGT | 5’-CACCGAATGGATTGGTCATCCTGGTC-3’  5’-AAACGACCAGGATGACCAATCCATTC-3’ |
| #2 | AAAGGTGGTCTATGTTGGCGTCTGGAT | 5’-CACCGAAAGGTGGTCTATGTTGGCGT-3’  5’-AAACACGCCAACATAGACCACCTTTC-3’ |
| #3 | CCGCTTCTACCCCAATGACTTGTGGGT | 5’-CACCGCCGCTTCTACCCCAATGACTT-3’  5’-AAACAAGTCATTGGGGTAGAAGCGGC-3’ |
| #4 | TCCTCCTGGAAATCATCAAGCAAGGGT | 5’-CACCGTCCTCCTGGAAATCATCAAGC-3’  5’-AAACGCTTGATGATTTCCAGGAGGAC-3’ |
| #5 | TGGAAATCATCAAGCAAGGGTGTGAGT | 5’-CACCGTGGAAATCATCAAGCAAGGGT-3’  5’-AAACACCCTTGCTTGATGATTTCCAC-3’ |
| #6 | ACTCCATCATCTTCTTAACTGGCATTG | 5’-CACCGCAATGCCAGTTAAGAAGATGA-3’  5’-AAACTCATCTTCTTAACTGGCATTGC-3’ |
| #7 | ACCCCAATGACTTGTGGGTGGTTGTGT | 5’-CACCGACACAACCACCCACAAGTCAT-3’  5’-AAACATGACTTGTGGGTGGTTGTGTC-3’ |
| #8 | ATCCTGCCTGGTATTGTCATCCTGTCC | 5’-CACCGAAGAAACTGAGAAGCATGA-3’  5’-AAACTCATGCTTCTCAGTTTCTTC-3’ |
| #9 | ATCCTGTCCTGCTATTGCATTATCATC | 5’-CACCGAAGCATGACGGACAAGTAC-3’  5’-AAACGTACTTGTCCGTCATGCTTC-3’ |
| #10 | ACTCCAAGGGCCACCAGAAGCGCAAGG | 5’-CACCGCCTTGCGCTTCTGGTGGCCCT-3’  5’-AAACAGGGCCACCAGAAGCGCAAGGC-3’ |
| #11 | ACTCCTTCATCCTCCTGGAAATCATCA | 5’-CACCGTGATGATTTCCAGGAGGATGA-3’  5’-AAACTCATCCTCCTGGAAATCATCAC-3’ |
| #12 | ATCCTCCTGGAAATCATCAAGCAAGGG | 5’-CACCGCCCTTGCTTGATGATTTCCAG-3’  5’-AAACCTGGAAATCATCAAGCAAGGGC-3’ |
| sg-CCR5 | ACTCTGCTTCGGTGTCGAAATGAGAAG | 5’-CACCGCTTCTCATTTCGACACCGAAG-3’  5’-AAACCTTCGGTGTCGAAATGAGAAG-3’ |

PAM sequences are indicated by red.
