# Supplementary figures and images for: Genome modification of CXCR4 by Staphylococcus aureus Cas9 renders cells resistance to HIV-1 infection
Source: Retrovirology. 2017 Nov 15;14:51. doi: 10.1186/s12977-017-0375-0 (PMC5688617; doi:10.1186/s12977-017-0375-0)

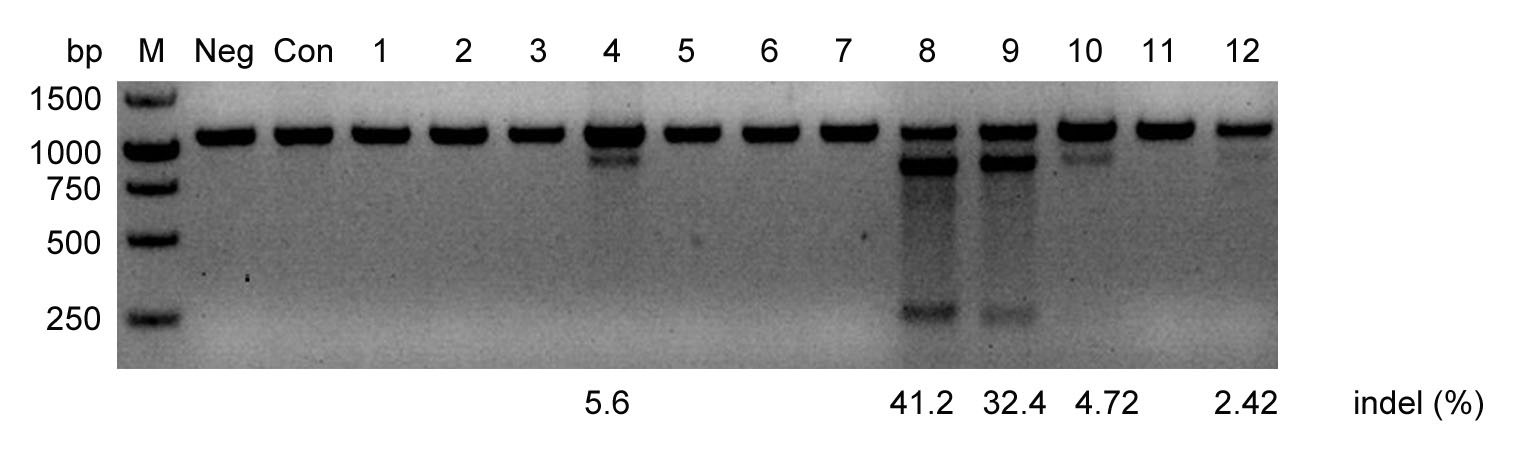

Supplement: Supplementary file 2 — Additional file 2: Figure S1. CXCR4 gene disruption screening analysis in HEK293T cells by the T7E1 cleavage assay. HEK293T cells were transfected in 24-well plates using 1 µg of plasmid DNA mixed with Polyethylenimine (PEI). Three days after the transfection, the genomic DNA was extracted and used as template to amplify a CXCR4 fragment (1100 bp). Neg: CCR5 sgRNA; Con: lentiviral vectors expressing SaCas9 only; #1–#12: lentiviral vectors expressing SaCas9/sgRNA #1–#12. [file 12977_2017_375_MOESM2_ESM.jpg]

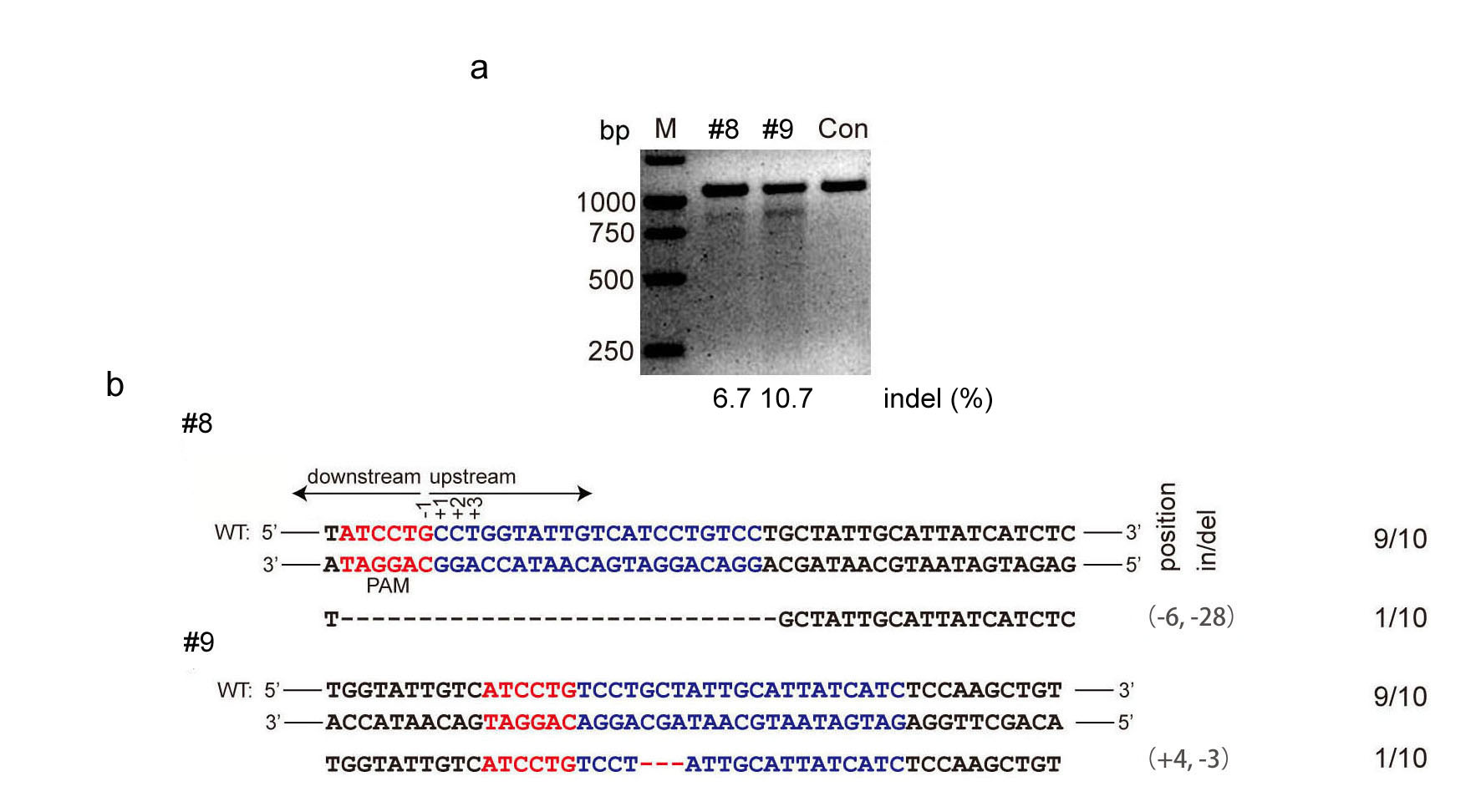

Supplement: Supplementary file 3 — Additional file 3: Figure S2. (a) T7E1 cleavage assay after electroporation in CD4+ T cells. (b) DNA sequences of CXCR4 of electroporated CD4+ T cells. [file 12977_2017_375_MOESM3_ESM.jpg]

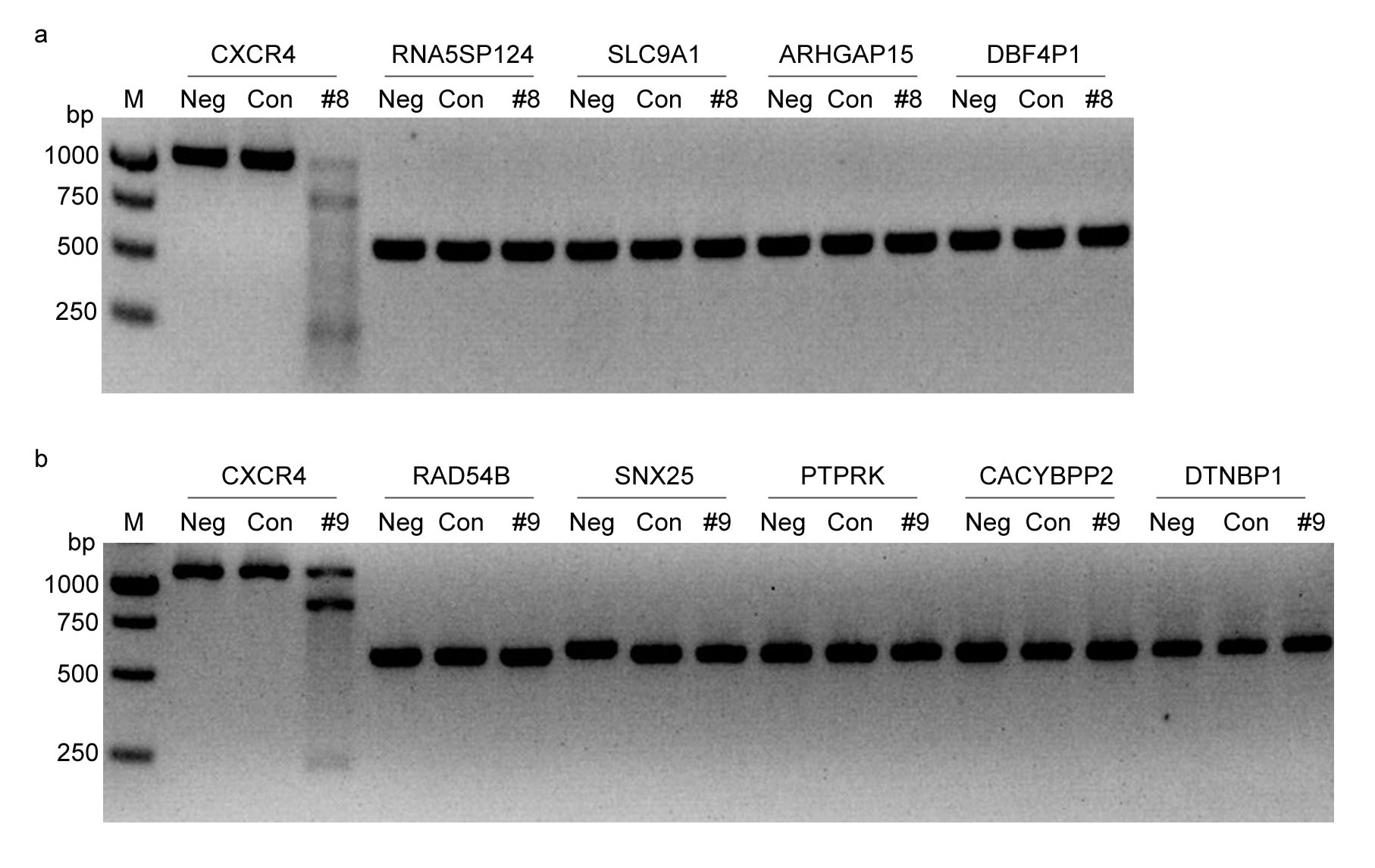

Supplement: Supplementary file 5 — Additional file 5: Figure S3. (a) Off-target analysis of CXCR4 (#8) by T7E1 cleavage assay. (b) Off-target analysis of CXCR4 (#9) by T7E1 cleavage assay. [file 12977_2017_375_MOESM5_ESM.jpg]
