# Supplementary material for: Genome modification of CXCR4 by Staphylococcus aureus Cas9 renders cells resistance to HIV-1 infection
Source: Retrovirology. 2017 Nov 15;14:51. doi: 10.1186/s12977-017-0375-0 (PMC5688617; doi:10.1186/s12977-017-0375-0)
Supplement: Supplementary file 4 — Additional file 4: Table S2. List of potential off-target sites for both 5′ and 3′ sgRNAs. [file 12977_2017_375_MOESM4_ESM.doc]

Table S2. List of potential off-target sites for both 5’ and 3’ sgRNAs

| sgRNA | Gene name | Predicted off-target sequence | Genome location | Coordinates | Mismatches |
| --- | --- | --- | --- | --- | --- |
| #8 | RNA5SP124 | tGcCAGGATGACAAaACCAGcAAGAAT | intergenic | Chr3: 14393322 | 4 |
|  | SLC9A1 | GGtgAGtATGACAAgACCAGGGTGAAT | intergenic | Chr1: 27095538 | 4 |
|  | ARHGAP15 | GGcCAGGAgttCAATACCAGGCTGGGT | intergenic | Chr2: 143077357 | 4 |
|  | DBF4P1 | aGACAGGAaGAaAATACCAGGCAGAGT | intergenic | Chr10: 64264186 | 3 |
| #9 | RAD54B | GAgtATAAaGCAATAGCAtGAGGGAGT | intronic | Chr8: 94418590 | 4 |
|  | SNX25 | GATGAaAcTGCAAaAaCAGGAATGGGT | intronic | Chr4: 185255883 | 4 |
|  | PTPRK | GcTGATAATGCAAcAGCgtGAATGGAT | intronic | Chr6:128234626 | 4 |
|  | CACYBPP2 | GgTGATAATGCAATAGtttGAATGAGT | intergenic | Chr2:183694296 | 4 |
|  | DTNBP1 | GATGATAATGCAAaAGCAatgATGAGT | intergenic | Chr6: 15707277 | 4 |

Mis-matched sequences are indicated by lowercase. PAM sequences are indicated by red. Chr, chromosome.
