# Supplementary material for: Genome modification of CXCR4 by Staphylococcus aureus Cas9 renders cells resistance to HIV-1 infection
Source: Retrovirology. 2017 Nov 15;14:51. doi: 10.1186/s12977-017-0375-0 (PMC5688617; doi:10.1186/s12977-017-0375-0)
Supplement: Supplementary file 6 — Additional file 6: Table S3. Primers information for the study. [file 12977_2017_375_MOESM6_ESM.docx]

Table S3. Primer information for the studies

| Primer | Sequences | Product size (bp) |
| --- | --- | --- |
| CXCR4-F | AGAATTTTGTAATTGGTTCTACCAAAG | 1100 |
| CXCR4-R | ACAACAGTGGAAGAAAGCTAGGGC |  |
| RNA5SP124-F | GTGCTGGGCCCGAGCCTTTCA | 597 |
| RNA5SP124-R | TCTGATCTGTGGTTTCTAGCC |  |
| SLC9A1-F | CCGTCTCTACTAAAAACACAA | 600 |
| SLC9A1-R | CACTGGCGAGTCCCAACTGAA |  |
| ARHGAP15-F | TGAGACAGGGTCCTGCTCTGT | 590 |
| ARHGAP15-R | GAGGTTGAATTGAGCCAAGAT |  |
| DBF4P1-F | TCAGAAAGAGCTCTCAAAATG | 610 |
| DBF4P1-R | GGCTAACAATAGCTTTTAAAT |  |
| RAD54B-F | ACCAGTCCAATGAATTTAAAG | 597 |
| RAD54B-R | GTTCATAGAAGCTTTATGATA |  |
| SNX25-F | CTTATCTTGTTCACCTATGTG | 600 |
| SNX25-R | TGATTCCAGCAGCCTCAGTTG |  |
| PTPRK-F | GGATAAAAACGCAAGTAATGC | 600 |
| PTPRK-R | GAGAAATTAAGACATGTGCCC |  |
| CACYBPP2-F | AGATTTACACAAATATGTTTT | 600 |
| CACYBPP2-R | ATAGATATCATAACAGTACTT |  |
| DTNBP1-F | GAATAATCAGAAAGTCATGGA | 597 |
| DTNBP1-R | GCCATGTTGCCCAGGCATGGG |  |
